# Radiologic Progression of Interstitial Lung Abnormalities following Surgical Resection in Patients with Lung Cancer

**DOI:** 10.3390/jcm12216858

**Published:** 2023-10-30

**Authors:** Yoon Joo Shin, Jeong Geun Yi, Mi Young Kim, Donghee Son, Su Yeon Ahn

**Affiliations:** 1 Department of Radiology, Konkuk University Medical Center, Konkuk University School of Medicine, Seoul 05030, Republic of Korea; yjshin87@gmail.com (Y.J.S.);; 2 Research Coordinating Center, Konkuk University Medical Center, Seoul 05030, Republic of Korea

**Keywords:** computed tomography, interstitial lung abnormalities, progression, lung cancer, surgical resection

## Abstract

In this study, we aimed to assess the prevalence of interstitial lung abnormalities (ILAs) and investigate the rates and risk factors associated with radiologic ILA progression among patients with lung cancer following surgical resection. Patients who underwent surgical resection for lung cancer at our institution from January 2015 to December 2020 were retrospectively evaluated and grouped according to their ILA status as having no ILAs, equivocal ILAs, or ILAs. Progression was determined by simultaneously reviewing the baseline and corresponding follow-up computed tomography (CT) scans. Among 346 patients (median age: 67 (interquartile range: 60–74) years, 204 (59.0%) men), 22 (6.4%) had equivocal ILAs, and 33 (9.5%) had ILAs detected upon baseline CT. Notably, six patients (6/291; 2.1%) without ILAs upon baseline CT later developed ILAs, and 50% (11/22) of those with equivocal ILAs exhibited progression. Furthermore, 75.8% (25/33) of patients with ILAs upon baseline CT exhibited ILA progression (76.9% and 71.4% with fibrotic and non-fibrotic ILAs, respectively). Multivariate analysis revealed that ILA status was a significant risk factor for ILA progression. ILAs and equivocal ILAs were associated with radiologic ILA progression after surgical resection in patients with lung cancer. Hence, early ILA detection can significantly affect clinical outcomes.

## 1. Introduction

Interstitial lung abnormalities (ILAs) refer to the incidental findings of computed tomography (CT) abnormalities without clinical suspicion of interstitial lung disease (ILD). They have been increasingly emphasized and recognized in recent years owing to their clinical impact, potential for progression to clinically significant ILD, and association with adverse clinical outcomes, such as all-cause mortality [1].

Old age and prolonged tobacco smoke exposure, which are both common among patients with idiopathic pulmonary fibrosis, along with other factors, such as occupational or environmental hazards and genetic predisposition, have been identified as risk factors for ILAs [2]. Overlapping risk factors highlight ILAs in patients with lung cancer. They are associated with an increased prevalence and mortality in populations with a high prevalence of smoking, such as lung cancer screening or chronic obstructive pulmonary disease populations, as well as in the general population [3,4,5,6,7]. Additionally, ILAs should be considered important in patients with lung cancer because of the increased risk of complications with cancer treatments, including surgery, chemotherapy, and radiotherapy [2,8,9,10]. The prevalence and clinical course of ILAs in patients with lung cancer need to be better understood to provide appropriate monitoring and improve patient prognosis.

Studies have been conducted on ILA progression in patient cohorts, including the National Lung Screening Trial (NLST) [3]; however, studies on ILA progression over time, specifically in patients with lung cancer, are lacking. This leaves a knowledge gap regarding radiologic progression and associated risk factors in this patient group.

Therefore, the aim of this study was to investigate the prevalence of ILAs and the rate and risk factors of radiologic ILA progression in patients with lung cancer following surgical resection. 

## 2. Materials and Methods

### 2.1. Study Population

**Figure 1:**
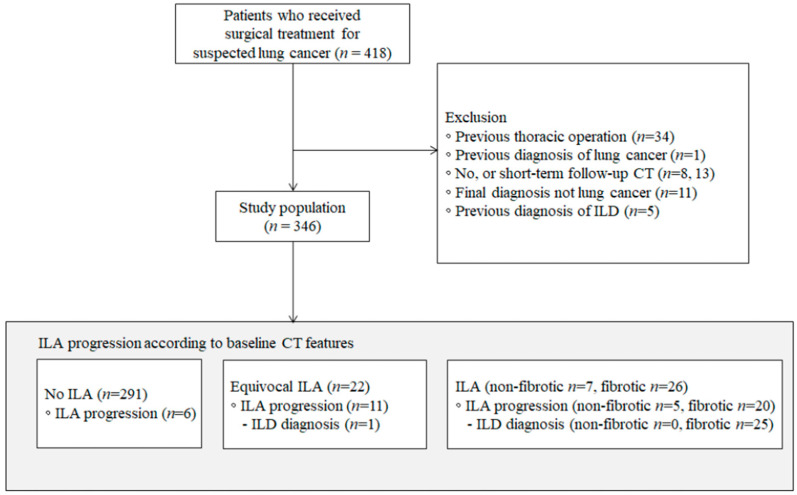
Flowchart of patient selection. CT, computed tomography; ILA, interstitial lung abnormality; ILD, interstitial lung disease.

Among the 418 consecutive patients who underwent surgical resection for lung cancer at our institution from January 2015 to December 2020, we excluded patients with a history of thoracic operations (*n* = 34), those with a previous diagnosis of lung cancer (*n* = 1), those without a follow-up CT (*n* = 8), those with only short-term follow-up CTs within 90 days (*n* = 13), those whose final diagnosis was not lung cancer (*n* = 11), and those with a previous diagnosis of ILD (*n* = 5). As a result, 346 patients were included in this study (Figure 1).

This retrospective study was approved by the Institutional Review Board of Konkuk University Medical Center. The requirement for written informed consent was waived owing to the retrospective nature of the study.

### 2.2. Data Collection

Patient-related demographics, including age, sex, smoking history, pulmonary function tests (the forced expiratory volume in the first second (FEV1), forced vital capacity (FVC), and FEV1/FVC ratio), tumor histology, stage, extent, and multiplicity of surgery, treatment options (chemotherapy and radiation therapy) and ILD diagnosis during follow-up, were collected from the electronic medical record system. Pathological staging was reevaluated according to the eighth edition of the tumor, node, and metastasis staging system. Anticancer drugs were classified as cytotoxic chemotherapy, oncogene-targeted agents, and immunotherapy.

### 2.3. CT Protocol

Chest CT scans were obtained using the following multidetector CT scanners, each with more than 16-section channels: Optima 660CT (GE Healthcare, Chicago, IL, USA), LightSpeed VCT (GE Healthcare), Revolution Apex (GE Healthcare), SOMATOM Definition (Siemens Healthineers, Erlangen, Germany), and SOMATOM Force (Siemens Healthineers). Images were obtained during breath-holding with full inspiration in the supine position. The slice thickness used at our hospital was 1.0–2.5 mm.

### 2.4. CT Image Evaluation

The baseline chest CT images obtained at the time of lung cancer diagnosis and the last follow-up chest CT images were retrospectively reviewed. Two thoracic radiologists (Y.J.S. and S.Y.A., with 5 and 8 years of experience in thoracic imaging, respectively), blinded to the clinical data, independently reviewed the CT images to determine the presence of ILAs. ILAs were scored using a 3-point scale (“0”, no evidence of ILAs; “1”, equivocal ILAs; and “2”, ILAs). Using a modified sequential reading method, each radiologist initially reviewed half of the total CT scans independently. After this initial review, they cross-read each other’s scans that were scored as either 1 or 2. Additionally, 20% of the scans that were initially scored as 0 by one radiologist were also cross-reviewed by the other [5,11,12].

ILAs were defined as the presence of any non-dependent abnormality affecting >5% of any lung zone, including ground glass or reticular abnormalities, architectural distortion, traction bronchiectasis, non-emphysematous cysts, and honeycombing (score 2) [2]. ILAs affecting <5% of any lung zone or that were unilateral were defined as equivocal ILAs (score 1). ILAs were further classified into the following two subcategories, modifying from the classification in the Position Paper by the Fleischner Society [2]: (i) fibrotic ILAs (ILAs with traction bronchiectasis, architectural distortion, and honeycombing, providing evidence of lung fibrosis) and (ii) nonfibrotic ILAs.

**Figure 2:**
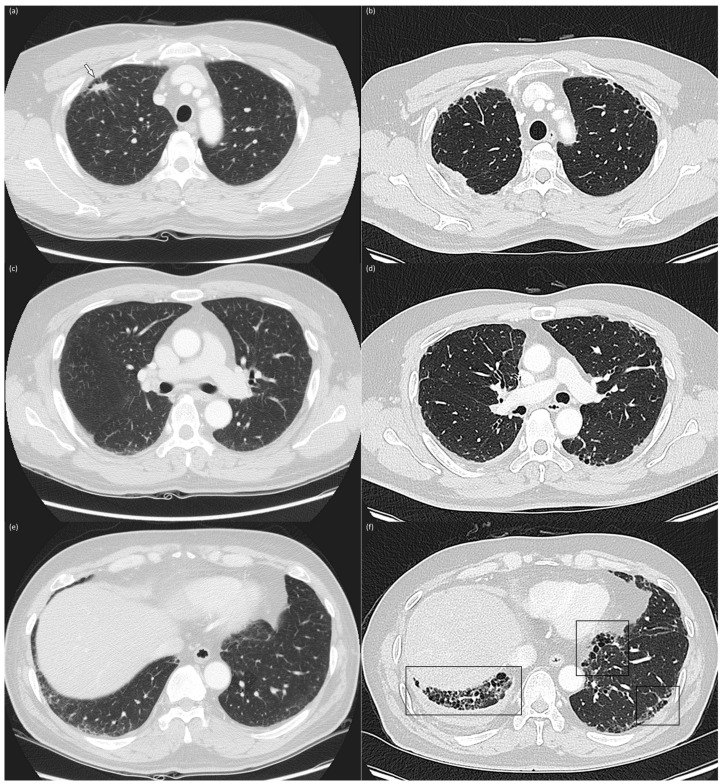
Baseline and 4-year follow-up chest computed tomography images Baseline (**a**,**c**,**e**) and 4-year follow-up (**b**,**d**,**f**) chest CT images in the upper (**a**,**b**), middle (**c**,**d**), and lower (**e**,**f**) lung zones of a 56-year-old, male current smoker with a 60 pack-year smoking history. The baseline CT reveals subpleural reticulation and mild traction bronchiolectasis in both lungs, suggesting the presence of fibrotic ILAs. A 1.5 cm-sized nodule (arrow) was identified in the right upper lobe and confirmed as adenocarcinoma (**a**). The patient underwent right upper lobe lobectomy and adjuvant chemotherapy with cisplatin and paclitaxel. The 4-year follow-up CT reveals increased subpleural reticulation, traction bronchiectasis, and honeycombing (box), predominantly in lower lung zones, indicating ILA progression.

The baseline and corresponding follow-up CT scans were simultaneously reviewed to determine progression [13]. Progression was defined as an increase in the lung area or a new appearance of an interstitial abnormality (Figure 2). Progression was assessed only in patients with an ILA score of 2 on the follow-up CT. In the case of a discrepancy between the two raters, the final decision was made by consensus.

The ILA status and lesion characteristics, including the lesion size and type (solid, part-solid, and ground glass opacity), were recorded.

### 2.5. Statistical Analysis

Patient demographics were compared between patients with ILAs and those with equivocal ILAs and no ILAs using the Wilcoxon rank-sum test for continuous variables and the chi-squared or Fisher’s exact tests for categorical variables. Post-hoc analysis was performed using the Bonferroni method; thus, *p*-values were calculated by dividing the raw *p*-values by the number of comparisons. Univariate and multivariate logistic regression analyses were performed to identify factors associated with ILA progression.

Variables with *p*-values < 0.5 in the univariate analyses were included in the multivariate analysis. *p*-values < 0.05 in the multivariate analysis were considered statistically significant. Cohen’s kappa agreement was used to assess the interrater agreement for each ILA status and progression. CT scans with an ILA score of 2 were analyzed for agreement with the type of ILA. Interrater agreement was classified as poor (κ = 0–0.20), fair (κ = 0.21–0.40), moderate (κ = 0.41–0.60), good (κ = 0.61–0.80), or excellent (κ = 0.81–1.00). The reported *p*-values are two-sided, and those <0.05 were considered statistically significant. Statistical analyses were performed using the R software (version 4.2.1; R Foundation for Statistical Computing, Vienna, Austria).

## 3. Results

### 3.1. Patient Demographics and ILA Status

The demographic characteristics and ILA status of patients at the baseline chest CT are summarized in Table 1 and Table S1. The median age of the study population was 67 (interquartile range (IQR): 60–74) years. Among the total 346 patients, 204 (59.0%) were men, and the former and current smokers constituted 110 (32.3%) and 85 (24.9%), respectively. Most patients were diagnosed with adenocarcinoma (79.8%), presented with stage I cancer (68.2%), and underwent lobectomy (71.4%). Of these patients, 166 (48.0%) and 33 (9.5%) received chemotherapy and radiotherapy, respectively.

Patients with ILAs (*n* = 33) were significantly older, more likely to be male, more likely to be a former or current smoker, more likely to have solid-type lung cancer, more likely to be diagnosed with squamous cell carcinoma or others, and less likely to receive oncogene-targeted therapy than patients without ILAs. Patients with equivocal ILAs (*n* = 22) were significantly older, more likely to have an FEV1/FVC ratio ≤70, and more likely to be diagnosed with squamous cell carcinoma or others than patients without ILAs. No significant differences were observed in operation type, cancer stage, radiotherapy, or follow-up period between the groups.

### 3.2. Factors Associated with ILA Progression

The median time between baseline and the last follow-up CT scan was 1313 (IQR: 945–1683) days. Six patients (6/291; 2.1%) without ILAs on baseline CT images developed ILAs, and 11 patients (11/22, 50.0%) with equivocal ILAs exhibited ILA progression. Twenty-five patients with ILAs on baseline CT images (25/33; 75.8%) exhibited ILA progression (20 of 26 [76.9%] patients with fibrotic ILAs and 5 of 7 [71.4%] with nonfibrotic ILAs). Among the entire cohort, six patients were diagnosed with ILD during the follow-up period: one with equivocal ILAs (1/22, 4.5%) and five with fibrotic ILAs (5/26, 19.2%).

Univariate and multivariate analyses of the associations between the clinical and radiologic factors and ILA progression are summarized in Table 2. Univariate analysis revealed that older age, male sex, former and current smoking, wedge and segmentectomy, solid lesion type, squamous cell carcinoma and others, adjuvant immunotherapy, and ILA status were significant risk factors for ILA progression. ILA status on baseline CT images was the only significant risk factor for ILA progression in the multivariate analysis (adjusted odds ratio (OR): 35.964, 95% confidence interval (CI): 10.033–147.398, *p* < 0.001 for equivocal ILAs; adjusted OR: 200.862, 95% CI: 22.823–4595.382, *p* < 0.001 for nonfibrotic ILAs; adjusted OR: 114.855, 95% CI: 29.377–561.518, *p* < 0.001 for fibrotic ILAs).

### 3.3. Interrater Agreement

Interrater agreement for the determination of the ILA status on baseline and follow-up CT scans was deemed good, with κ values of 0.717 and 0.787, respectively. For the categorization of ILA type, the agreement was excellent for baseline CT (κ = 0.904) and moderate for follow-up CT (κ = 0.574) images. The agreement for assessment of ILA progression was moderate (κ = 0.509).

## 4. Discussion

In this retrospective study, we assessed the prevalence and radiologic progression of ILAs in patients with lung cancer who underwent surgical resection. The study demonstrated that approximately 6.4% (22/346) of patients had equivocal ILAs, and 9.5% (33/346) had ILAs on baseline CT images. Furthermore, 2.1% (6/291), 50.0% (11/22), and 75.8% (25/33) of patients with no ILAs, equivocal ILAs, and ILAs, respectively, on CT images at the time of lung cancer diagnosis were observed to have ILA progression on the final follow-up CT image. Only the ILA status on baseline CT images was associated with radiologic ILA progression.

The prevalence of ILAs in the present study was 9.5%. This is consistent with a previous study by Hida et al., who reported a 9.5% prevalence among patients with stage I non-small cell lung cancer (NSCLC) [11]. Other large cohort studies or lung cancer screening cohorts have revealed a 7–10% prevalence [1,3]. However, the ILA prevalence among patients with lung cancer varies from 3.9% among patients with stage IV NSCLC, as reported by Araki et al. [14], to 21.7% among patients with stage I and II lung cancer, as reported by Iwasawa et al. [15]. Nishino et al. [16] reported an ILA prevalence of 14% among patients with stage IV NSCLC. Several factors could account for these variations in ILA prevalence. Different studies focus on patients with varying stages and types of lung cancer, which could influence ILA prevalence. The proportion of smokers in the study cohort can vary, potentially affecting ILA rates. Other factors such as age, gender, comorbidities, and environmental exposures might also have influence ILA prevalence. Additionally, the diagnosis of ILA can be subjective, leading to potential variability in interpretation among radiologists.

To the best of our knowledge, this is the first study in which ILA progression was investigated in patients with lung cancer. ILAs have garnered much attention in patients with lung cancer owing to their potential impact on treatment outcomes and mortality. They are associated with an increased risk of postoperative complications, immune-related pneumonitis, and radiation pneumonitis, as well as increased mortality [8,9,10,15,17]. However, a gap exists in the understanding of the natural course of radiologic ILA progression.

Several studies have been conducted to investigate ILA progression rates in large cohorts and lung cancer screening populations. Patel et al. reported that 24.4% (10/41) of patients with ILAs on baseline CT images in a lung cancer screening cohort were later diagnosed with ILD [18]. The progression of ILAs was estimated to be approximately 20% (16/79) over 2 years in the NLST and 25% (3/12) with other chronic interstitial pneumonia patterns over 3 years in the Multicentric Italian Lung Detection trials [3,19]. With a longer follow-up period, >40% of ILAs progress over 5 years [6]. A study involving an Asian health screening cohort revealed an ILA progression rate of 80% (48/60) over 8 years [5]. However, the natural course of ILAs after treating patients with lung cancer remains unclear. The present study uniquely addresses this gap by offering clinicians insights that can influence pre-treatment risk stratification and postoperative follow-up planning, potentially affecting treatment outcomes.

The progression rates observed in our study population seemed higher than those previously reported in general or lung cancer screening cohorts. We hypothesized that patients with lung cancer may represent a high-risk group for ILA progression owing to their exposure to various treatment methods, such as surgery, chemotherapy, and radiotherapy, which may accelerate ILA progression. Given the study’s methodology, classifying even slight changes as progression, the progression rate might have been overestimated. Nonetheless, the subsequent ILD diagnoses in 19.2% of the patients with fibrotic ILAs remain a clinically significant finding. Given the notable progression rates observed, it would be advisable for clinicians to include a focused evaluation of ILA progression during the follow-up assessments, especially those with baseline ILA. Furthermore, by educating patients about the potential risks of ILA progression, they can be better prepared to promptly report any new or worsening respiratory symptoms.

This study had several limitations. First, a selection bias might have occurred owing to the study’s retrospective nature. Second, the correlation between the radiologic progression of ILAs and clinical symptoms, such as dyspnea or decreased lung function, during the follow-up period was not investigated. Many patients did not undergo regular pulmonary function tests after surgery, and the documentation of patients’ symptoms in the electronic medical records (EMR) was inconsistent. Recognizing this, we believe that future research in this area would benefit from prospective studies that ensure accurate and consistent recording of patients’ clinical symptoms and outcomes. Third, only qualitative image analyses were performed. Hence, the degree of radiologic progression could not be adequately assessed. Consequently, even mild progression, which does not necessarily indicate clinically significant disease progression, might have been classified as progression. Additionally, ILA scoring may vary among radiologists. Further studies with quantitative analysis may provide better reproducibility and more detailed assessments of ILA progression. Furthermore, a systematic evaluation of the correlation between clinical symptoms and radiologic features is warranted to determine the impact of ILAs on patient outcomes.

In conclusion, the ILAs and equivocal ILAs detected on CT images at lung cancer diagnosis were associated with ILA progression after surgical resection for lung cancer. Hence, early identification of ILAs and an understanding of their implications are essential, and heightened awareness and monitoring are recommended to ensure optimal patient care and management.

## Figures and Tables

**Table 1:** Baseline characteristics and clinical events according to ILA status of patients (*n* = 346) and ILA progression rates.

	ILA Status on Baseline CT					
Variables	Total (*n* = 346)	No ILA (*n* = 291)	Equivocal ILA (*n* = 22)	*p*	ILA (*n* = 33)	*p*
Age	67 (60.25, 74)	66 (59, 72)	76.5 (73, 77)	<0.001	75 (69, 78)	<0.001
Sex						
M	204 (59.0)	161 (55.3)	14 (63.6)	0.449	29 (87.9)	<0.001
F	142 (41.0)	130 (44.7)	8 (36.4)		4 (12.1)	
Smoking (*n* = 341)						
Never	146 (42.8)	136 (47.4)	6 (27.3)	0.085	4 (12.5)	0.001
Former	110 (32.3)	82 (28.6)	11 (50.0)		17 (53.1)	
Current	85 (24.9)	69 (24.0)	5 (22.7)		11 (34.4)	
Operation type						
Lobectomy	247 (71.4)	216 (74.2)	13 (59.1)	0.187	18 (54.5)	0.051
Pneumonectomy	5 (1.4)	3 (1.0)	1 (4.5)		1 (3.0)	
Segmentectomy	4 (1.2)	4 (1.4)	0 (0.0)		0 (0.0)	
Wedge	90 (26.0)	68 (23.4)	8 (36.4)		14 (42.4)	
No. of operations						
1	320 (92.5)	270 (92.8)	22 (100.0)	0.380	28 (84.8)	0.164
≥2	26 (7.5)	21 (7.2)	0 (0.0)		5 (15.2)	
Size (cm)	2.3 (1.7, 3.3)	2.4 (1.7, 3.25)	2.1 (1.83, 2.77)	0.937	2.2 (1.5, 3.5)	0.733
Nodule type						
Solid	198 (57.2)	156 (53.6)	16 (72.7)	0.160	26 (78.8)	0.017
PSN	126 (36.4)	115 (39.5)	6 (27.3)		5 (15.2)	
Pure GGN	22 (6.4)	20 (6.9)	0 (0.0)		2 (6.1)	
Diagnosis						
ADC	276 (79.8)	248 (85.2)	14 (63.6)	0.018	14 (42.4)	<0.001
SQCC	57 (16.5)	35 (12.0)	6 (27.3)		16 (48.5)	
Others	13 (3.8)	8 (2.7)	2 (9.1)		3 (9.1)	
Stage						
1	236 (68.2)	194 (66.7)	16 (72.7)	0.114	26 (78.8)	0.194
2	42 (12.1)	32 (11.0)	5 (22.7)		5 (15.2)	
3	34 (9.8)	33 (11.3)	0 (0.0)		1 (3.0)	
4	34 (9.8)	32 (11.0)	1 (4.5)		1 (3.0)	
Anticancer drugs						
No	180 (52.0)	152 (52.2)	12 (54.5)	0.834	16 (48.5)	0.683
Yes	166 (48.0)	139 (47.8)	10 (45.5)		17 (51.5)	
Radiotherapy						
No	313 (90.5)	266 (91.4)	20 (90.9)	1.000	27 (81.8)	0.109
Yes	33 (9.5)	25 (8.6)	2 (9.1)		6 (18.2)	
ILD diagnosis						
No	340 (98.3)	291 (100.0)	21 (95.5)	0.070	28 (84.8)	<0.001
Yes	6 (1.7)	0 (0.0)	1 (4.5)		5 (15.2)	
ILA progression						
Stable	304 (87.9)	285 (97.9)	11 (50.0)	<0.001	8 (24.2)	<0.001
Progression	42 (12.1)	6 (2.1)	11 (50.0)		25 (75.8)	
Follow-up interval (days)	1312.5 (944.5, 1682.75)	1316 (944, 1702)	1389.5 (1103.25, 1508)	0.699	1254 (913, 1657)	0.443

Continuous values are presented as medians (Q1, Q3) and tested using the Wilcoxon rank-sum test. Categorical values are presented as *n* (%) and tested using the chi-squared or Fisher’s exact tests. *p* < 0.025 for statistical significance, taking into account Bonferroni correction by a number of comparisons (no ILAs vs. equivocal ILAs and no ILAs vs. ILAs) (0.05/2). ILA, interstitial lung abnormality; CT, computed tomography; M, male; F, female; ILD, interstitial lung disease; PY, pack-years; FVC, forced vital capacity; FEV1, forced expiratory volume in the first second; PSN, part-solid nodule; GGN, ground glass nodule; ADC, adenocarcinoma; SQCC, squamous cell carcinoma.

**Table 2:** Risk factors for ILA progression in patients with lung cancer after surgical resection.

	Univariate Analysis			Multivariate Analysis (*n* = 341)		
	Unadjusted OR	95% CI	*p*	Adjusted OR	95% CI	*p*
Age	1.101	1.055–1.153	<0.001	1.024	0.952–1.109	0.548
Sex						
M	Ref.			Ref.		
F	0.351	0.153–0.729	0.008	1.736	0.278–11.899	0.563
Smoking						
Never	Ref.			Ref.		
Former	3.885	1.623–10.333	0.004	1.895	0.275–13.109	0.513
Current	4.605	1.873–12.465	0.001	4.497	0.534–45.689	0.182
PY	1.018	1.006–1.031	0.003			
0	Ref.					
0 < PY ≤ 30	3.919	1.581–10.652	0.004			
>30	5.030	2.107–13.378	0.001			
Operation type						
Lobectomy+ Pneumonectomy	Ref.			Ref.		
Wedge+ Segmentectomy	2.005	1.014–3.889	0.041	1.273	0.409–3.891	0.671
No. of operation						
1	Ref.					
≥2	1.349	0.380–3.760	0.599			
Size	0.994	0.759–1.270	0.963			
Nodule type						
Solid	Ref.			Ref.		
PSN	0.399	0.174–0.835	0.020	1.295	0.372–4.557	0.682
Pure GGN	0.247	0.014–1.248	0.179	0.000	0.000–Inf	0.989
Diagnosis						
ADC	Ref.			Ref.		
SQCC	6.243	3.008–12.992	<0.001	1.543	0.409–5.734	0.516
Others	8.454	2.364–27.991	0.001	3.914	0.445–31.152	0.209
Stage						
1	Ref.					
2	1.025	0.366–2.472	0.958			
3	0.186	0.010–0.914	0.104			
4	0.384	0.061–1.353	0.204			
Anticancer drugs						
No	Ref.					
Yes	1.222	0.640–2.347	0.543			
Adjuvant cytotoxic chemotherapy						
No anticancer drugs	Ref.					
Yes	1.291	0.656–2.514	0.453			
No	0.680	0.105–2.523	0.617			
Adjuvant oncogene-targeted						
No anticancer drugs	Ref.					
Yes	0.434	0.100–1.316	0.189			
No	1.704	0.848–3.379	0.128			
Adjuvant immunotherapy						
No anticancer drugs	Ref.			Ref.		
Yes	2.736	0.982–6.992	0.042	2.808	0.481–15.493	0.241
No	0.907	0.429–1.853	0.793	1.181	0.357–3.890	0.782
Radiotherapy						
No	Ref.					
Yes	1.710	0.606–4.182	0.269			
Baseline ILA status						
No ILA	Ref.			Ref.		
Equivocal ILA	47.500	15.405–162.218	<0.001	35.964	10.033–147.398	<0.001
Nonfibrotic ILA	118.750	21.309–961.923	<0.001	200.862	22.823–4595.382	<0.001
Fibrotic ILA	158.333	50.517–592.398	<0.001	114.855	29.377–561.518	<0.001

Variables with *p*-values < 0.5 in the univariate analyses were included in the multivariable analysis. OR, odds ratio; CI, confidence interval; Ref., reference.

## Data Availability

The datasets generated during and/or analyzed during the current study are available from the corresponding author upon reasonable request.

## Supplementary Materials

The following supporting information can be downloaded at: https://www.mdpi.com/article/10.3390/jcm12216858/s1. Table S1. Baseline characteristics and clinical events according to ILA status of patients (*n* = 346) and ILA progression rates with detailed information.
