# Supplementary material for: Radiologic Progression of Interstitial Lung Abnormalities following Surgical Resection in Patients with Lung Cancer
Source: J Clin Med. 2023 Oct 30;12(21):6858. doi: 10.3390/jcm12216858 (PMC10647667; doi:10.3390/jcm12216858)
Supplement: Supplementary file 1 [file jcm-12-06858-s001.zip › jcm-2638013-supplementary.pdf]

**Table S1.** Baseline characteristics and clinical events according to ILA status of patients (N=346) and ILA progression rates with detailed information.

| Variables | ILA Status on Baseline CT | | | | | | | | | |
|---|---|---|---|---|---|---|---|---|---|---|
| | Total (N=346) | No ILA (N=291) | Equivocal ILA (N=22) | *P**, ** | Nonfibrotic ILA (N=7) | *P** | Fibrotic ILA (N=26) | *P** | ILA (N=33) | *P*** |
| Age | 67 (60.25, 74) | 66 (59, 72) | 76.5 (73, 77) | <0.001 | 70 (66, 77) | 0.153 | 75.5 (70.5, 79.5) | <0.001 | 75 (69, 78) | <0.001 |
| Sex | | | | | | | | | | |
| M | 204 (59.0) | 161 (55.3) | 14 (63.6) | 0.449 | 4 (57.1) | 1.000 | 25 (96.2) | <0.001 | 29 (87.9) | <0.001 |
| F | 142 (41.0) | 130 (44.7) | 8 (36.4) | | 3 (42.9) | | 1 (3.8) | | 4 (12.1) | |
| Smoking (N=341) | | | | | | | | | | |
| Never | 146 (42.8) | 136 (47.4) | 6 (27.3) | 0.085 | 2 (28.6) | 0.421 | 2 (8.0) | <0.001 | 4 (12.5) | 0.001 |
| Former | 110 (32.3) | 82 (28.6) | 11 (50.0) | | 2 (28.6) | | 15 (60.0) | | 17 (53.1) | |
| Current | 85 (24.9) | 69 (24.0) | 5 (22.7) | | 3 (42.9) | | 8 (32.0) | | 11 (34.4) | |
| Operation type | | | | | | | | | | |
| Lobectomy | 247 (71.4) | 216 (74.2) | 13 (59.1) | 0.187 | 3 (42.9) | 0.047 | 15 (57.7) | 0.201 | 18 (54.5) | 0.051 |
| Pneumonectomy | 5 (1.4) | 3 (1.0) | 1 (4.5) | | 1 (14.3) | | 0 (0.0) | | 1 (3.0) | |
| Segmentectomy | 4 (1.2) | 4 (1.4) | 0 (0.0) | | 0 (0.0) | | 0 (0.0) | | 0 (0.0) | |
| Wedge | 90 (26.0) | 68 (23.4) | 8 (36.4) | | 3 (42.9) | | 11 (42.3) | | 14 (42.4) | |
| No. of operations | | | | | | | | | | |
| 1 | 320 (92.5) | 270 (92.8) | 22 (100.0) | 0.380 | 7 (100.0) | 1.000 | 21 (80.8) | 0.049 | 28 (84.8) | 0.164 |
| ≥2 | 26 (7.5) | 21 (7.2) | 0 (0.0) | | 0 (0.0) | | 5 (19.2) | | 5 (15.2) | |
| Size (cm) | 2.3 (1.7, 3.3) | 2.4 (1.7, 3.25) | 2.1 (1.83, 2.77) | 0.937 | 2.2 (1.7, 3.4) | 0.890 | 2.3 (1.5, 3.45) | 0.749 | 2.2 (1.5, 3.5) | 0.733 |
| Nodule type | | | | | | | | | | |
| Solid | 198 (57.2) | 156 (53.6) | 16 (72.7) | 0.160 | 5 (71.4) | 0.238 | 21 (80.8) | 0.028 | 26 (78.8) | 0.017 |
| PSN | 126 (36.4) | 115 (39.5) | 6 (27.3) | | 1 (14.3) | | 4 (15.4) | | 5 (15.2) | |
| Pure GGN | 22 (6.4) | 20 (6.9) | 0 (0.0) | | 1 (14.3) | | 1 (3.8) | | 2 (6.1) | |
| Diagnosis | | | | | | | | | | |
| ADC | 276 (79.8) | 248 (85.2) | 14 (63.6) | 0.018 | 4 (57.1) | 0.083 | 10 (38.5) | <0.001 | 14 (42.4) | <0.001 |
| SQCC | 57 (16.5) | 35 (12.0) | 6 (27.3) | | 3 (42.9) | | 13 (50.0) | | 16 (48.5) | |
| Others | 13 (3.8) | 8 (2.7) | 2 (9.1) | | 0 (0.0) | | 3 (11.5) | | 3 (9.1) | |
| Stage | | | | | | | | | | |
| 1 | 236 (68.2) | 194 (66.7) | 16 (72.7) | 0.114 | 5 (71.4) | 0.469 | 21 (80.8) | 0.461 | 26 (78.8) | 0.194 |
| 2 | 42 (12.1) | 32 (11.0) | 5 (22.7) | | 2 (28.6) | | 3 (11.5) | | 5 (15.2) | |

| | | | | | | | | | | |
|---|---|---|---|---|---|---|---|---|---|---|
| 3 | 34 (9.8) | 33 (11.3) | 0 (0.0) | | 0 (0.0) | | 1 (3.8) | | 1 (3.0) | |
| 4 | 34 (9.8) | 32 (11.0) | 1 (4.5) | | 0 (0.0) | | 1 (3.8) | | 1 (3.0) | |
| Anticancer drugs | | | | | | | | | | |
| No | 180 (52.0) | 152 (52.2) | 12 (54.5) | 0.834 | 2 (28.6) | 0.269 | 14 (53.8) | 0.875 | 16 (48.5) | 0.683 |
| Yes | 166 (48.0) | 139 (47.8) | 10 (45.5) | | 5 (71.4) | | 12 (46.2) | | 17 (51.5) | |
| Neoadjuvant cytotoxic chemotherapy | | | | | | | | | | |
| No anticancer drugs | 319 (92.2) | 270 (92.8) | 21 (95.5) | 0.460 | 4 (57.1) | 0.013 | 24 (92.3) | 0.800 | 28 (84.8) | 0.149 |
| Yes | 18 (5.2) | 14 (4.8) | 0 (0.0) | | 2 (28.6) | | 2 (7.7) | | 4 (12.1) | |
| No | 9 (2.6) | 7 (2.4) | 1 (4.5) | | 1 (14.3) | | 0 (0.0) | | 1 (3.0) | |
| Neoadjuvant oncogene-targeted | | | | | | | | | | |
| No anticancer drugs | 319 (92.2) | 270 (92.8) | 21 (95.5) | 0.625 | 4 (57.1) | 0.004 | 24 (92.3) | 0.436 | 28 (84.8) | 0.037 |
| Yes | 10 (2.9) | 9 (3.1) | 1 (4.5) | | 0 (0.0) | | 0 (0.0) | | 0 (0.0) | |
| No | 17 (4.9) | 12 (4.1) | 0 (0.0) | | 3 (42.9) | | 2 (7.7) | | 5 (15.2) | |
| Neoadjuvant immunotherapy | | | | | | | | | | |
| No anticancer drugs | 319 (92.2) | 270 (92.8) | 21 (95.5) | 1.000 | 4 (57.1) | 0.008 | 24 (92.3) | 0.749 | 28 (84.8) | 0.17 |
| Yes | 4 (1.2) | 3 (1.0) | 0 (0.0) | | 1 (14.3) | | 0 (0.0) | | 1 (3.0) | |
| No | 23 (6.6) | 18 (6.2) | 1 (4.5) | | 2 (28.6) | | 2 (7.7) | | 4 (12.1) | |
| Adjuvant cytotoxic chemotherapy | | | | | | | | | | |
| No anticancer drugs | 194 (56.1) | 162 (55.7) | 13 (59.1) | 0.842 | 3 (42.9) | 0.684 | 16 (61.5) | 0.709 | 19 (57.6) | 0.594 |
| Yes | 127 (36.7) | 106 (36.4) | 8 (36.4) | | 4 (57.1) | | 9 (34.6) | | 13 (39.4) | |
| No | 25 (7.2) | 23 (7.9) | 1 (4.5) | | 0 (0.0) | | 1 (3.8) | | 1 (3.0) | |
| Adjuvant oncogene-targeted | | | | | | | | | | |
| No anticancer drugs | 194 (56.1) | 162 (55.7) | 13 (59.1) | 0.509 | 3 (42.9) | 0.591 | 16 (61.5) | 0.041 | 19 (57.6) | 0.044 |
| Yes | 57 (16.5) | 54 (18.6) | 2 (9.1) | | 1 (14.3) | | 0 (0.0) | | 1 (3.0) | |
| No | 95 (27.5) | 75 (25.8) | 7 (31.8) | | 3 (42.9) | | 10 (38.5) | | 13 (39.4) | |
| Adjuvant immunotherapy | | | | | | | | | | |
| No anticancer drugs | 194 (56.1) | 162 (55.7) | 13 (59.1) | 0.388 | 3 (42.9) | 0.450 | 16 (61.5) | 0.452 | 19 (57.6) | 0.464 |
| Yes | 27 (7.8) | 20 (6.9) | 3 (13.6) | | 1 (14.3) | | 3 (11.5) | | 4 (12.1) | |
| No | 125 (36.1) | 109 (37.5) | 6 (27.3) | | 3 (42.9) | | 7 (26.9) | | 10 (30.3) | |
| Radiotherapy | | | | | | | | | | |
| No | 313 (90.5) | 266 (91.4) | 20 (90.9) | 1.000 | 6 (85.7) | 0.476 | 21 (80.8) | 0.085 | 27 (81.8) | 0.109 |
| Yes | 33 (9.5) | 25 (8.6) | 2 (9.1) | | 1 (14.3) | | 5 (19.2) | | 6 (18.2) | |
| ILD diagnosis | | | | | | | | | | |
| No | 340 (98.3) | 291 (100.0) | 21 (95.5) | 0.070 | 7 (100.0) | - | 21 (80.8) | <0.001 | 28 (84.8) | <0.001 |

| | | | | | | | | | | |
|---|---|---|---|---|---|---|---|---|---|---|
| Yes | 6 (1.7) | 0 (0.0) | 1 (4.5) | | 0 (0.0) | | 5 (19.2) | | 5 (15.2) | |
| ILA progression | | | | | | | | | | |
| Stable | 304 (87.9) | 285 (97.9) | 11 (50.0) | <0.001 | 2 (28.6) | <0.001 | 6 (23.1) | <0.001 | 8 (24.2) | <0.001 |
| Progression | 42 (12.1) | 6 (2.1) | 11 (50.0) | | 5 (71.4) | | 20 (76.9) | | 25 (75.8) | |
| Follow-up interval (days) | 1312.5 (944.5, 1682.75) | 1316 (944, 1702) | 1389.5 (1103.25, 1508) | 0.699 | 1654 (1200, 1816) | 0.419 | 1071 (810.25, 1627.75) | 0.2 | 1254 (913, 1657) | 0.443 |

Continuous values are presented as medians (Q1, Q3) and tested using the Wilcoxon rank-sum test. Categorical values are presented as n (%) and tested using the chi-squared or Fisher's exact tests. * *P*<0.017 for statistical significance, taking into account Bonferroni correction by a number of comparisons (no ILAs vs. equivocal ILAs, no ILAs vs. nonfibrotic ILAs, and no ILAs vs. fibrotic ILAs) (0.05/3). ** *P*<0.025 for statistical significance, taking into account Bonferroni correction by a number of comparisons (no ILAs vs. equivocal ILAs and no ILAs vs. ILAs) (0.05/2). *ILA*, interstitial lung abnormality; *CT*, computed tomography; *M*, male; *F*, female; *ILD,* interstitial lung disease; *PY*, pack-years; *FVC*, forced vital capacity; *FEV1*, forced expiratory volume in the first second; *PSN*, part-solid nodule; *GGN*, ground-glass nodule; *ADC,* adenocarcinoma; *SQCC*, squamous cell carcinoma
